# Prevalence of Anemia and Associated Factors in Child Bearing Age Women in Riyadh, Saudi Arabia

**DOI:** 10.1155/2013/636585

**Published:** 2013-09-24

**Authors:** AlJohara M. AlQuaiz, Ashry Gad Mohamed, Tawfik A. M. Khoja, Abdullah AlSharif, Shaffi Ahamed Shaikh, Hamad Al Mane, Abdallah Aldiris, Ambreen Kazi, Durdana Hammad

**Affiliations:** ^1^Princess Nora Bint Abdullah Chair for Women's Health Research, Research Chair Program, King Saud University, P.O. Box 231831, Riyadh 11321, Saudi Arabia; ^2^Department of Family & Community Medicine, College of Medicine, King Saud University, Riyadh, Saudi Arabia; ^3^Gulf Ministry of Health, Riyadh, Saudi Arabia; ^4^Council of Cooperative Health Insurance, Ministry of Health, Riyadh, Saudi Arabia; ^5^Ministry of Health, Riyadh, Saudi Arabia; ^6^Central Hospital Laboratory, Ministry of Health, Riyadh, Saudi Arabia

## Abstract

*Objective*. To determine the prevalence and risk factors for anemia in child bearing age women in Riyadh, Saudi Arabia. *Design*. Cross-sectional survey was conducted using two-stage cluster sampling. 25 clusters (primary health care centers (PHCC)) were identified from all over Riyadh, and 45–50 households were randomly selected from each cluster. Eligible women were invited to PHCC for questionnaire filling, anthropometric measurements, and complete blood count. Blood hemoglobin was measured with Coulter Cellular Analysis System using light scatter method. *Setting*. PHCC. *Subjects*. 969 (68%) women out of 1429 women were included in the analysis. *Results*. Mean hemoglobin was 12.35 (±1.80) g/dL, 95% CI 12.24–12.46 with interquartile range of 1.9. Anemia (Hb <12 g/dL) was present in 40% (390) women. Mean (±SD) for MCH, MCV, MCHC, and RDW was 79.21 (±12.17) fL, 26.37 (±6.21) pg, 32.36 (±4.91) g/dL, and 14.84 (±4.65)%, respectively. Multivariate logistic regression revealed that having family history of iron deficiency anemia (OR 2.91, 95% CI 1.78–4.76) and infrequent intake of meat (OR 1.54, 95%CI 1.15–2.05) were associated with increased risk of anemia, whereas increasing body mass index (OR 0.95, 95% CI 0.92–0.97) was associated with reduced risk of anemia. *Conclusion*. Women should be educated about proper diet and reproductive issues in order to reduce the prevalence of anemia in Saudi Arabia.

## 1. Introduction

Anemia is a public health issue for developing countries, especially for child bearing age women [1]. The worldwide prevalence of anemia in child bearing age group is quite high (30.2%) [1]. According to World Health Organization (WHO) report, 32.3% nonpregnant women of child bearing age are suffering from anemia in Saudi Arabia [1]. AlQuaiz conducted a hospital-based study and found 37% of women suffering from anemia in Riyadh, Saudi Arabia [2].

Anemia is a multifactor disease and can act both as a risk factor or a consequence of a disease [3]. There are various modifiable and nonmodifiable factors affecting anemia in combination or alone. These may range from ethnicity, gender, age, sociodemographic status, dietary habits, physical and mental health, gynecological/obstetric history, cancers, and anticancerous drugs to genetic makeup [3]. Specific risk factors include deficiency of iron, worm infestation, repeated pregnancies, menorrhagia, postpartum hemorrhage, gastric ulcers, hemorrhoids, intake of aspirin/nonsteroidal anti-inflammatory drugs, and pure vegetarian diet [3]. Controversial results from long- and short-term intervention studies on the role of different food inhibitors (tea, coffee, bran, and egg yolk) and enhancers (meat, and dairy products, and ascorbic acid) on iron absorption and iron stores have raised questions regarding their effectiveness in reducing anemia [4, 5].

Functional consequences of anemia are diverse. It follows the life cycle approach where each stage of life is affected. Inside the mother's womb it decreases fetus' physical growth and may cause mental growth retardation; during childhood it affects cognitive growth and development; during adult hood it decreases physical work capacity, and finally in elderly it affects the quality of life [6–13]. In addition, in severe cases it may lead to maternal and infant mortality [9–12].

Limited data is available on anemia from Saudi Arabia [14–16]. AlQuaiz found that dietary habits (infrequent intake of meat and juices), menorrhagia, intake of antacids, and nonsteroidal anti-inflammatory drugs were the most frequent correlates of anemia in Saudi women of child bearing age [2]. Institution/hospital-based studies are available [2, 14, 16] regarding prevalence and risk factors for anemia but are subjected to selection bias and may not be generalizable; therefore, reassessing anemia through primary care/community-based approach is a crucial component of public health research. The objective of present survey was to measure the prevalence and risk factors for anemia in women of child bearing age in Riyadh, Saudi Arabia.

## 2. Materials and Methods

### 2.1. Study Design and Setting

It was a community-based household cross-sectional survey. This survey covered whole Riyadh region with a population of 3,726,523 Saudi persons (Ministry of Planning, preliminary results of 1425 Hijri (2004) census). The study was approved by the Institutional Review Board, King Saud University, and funded by Ministry of Health, Kingdom of Saudi Arabia.

### 2.2. Sampling

**Figure 1:**
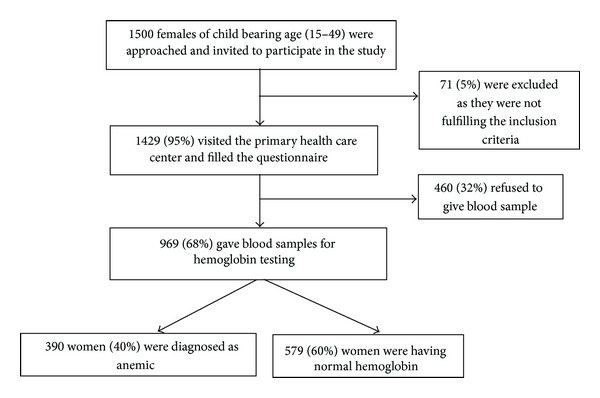
Enrollment flow chart of child bearing age women in Riyadh, Saudi Arabia.

Sample size was calculated on the assumption of 32% prevalence of iron deficiency anemia (IDA) in Riyadh. At 95% confidence level and acceptance of 3% as degree of precision, sample size was calculated as 500 child bearing age women. We used two-stage cluster sampling technique along with a design effect of 2 which increased the sample size to 1000 women. We expected 20% refusal rate which further increased the sample size to 1,200 women. For risk factor analysis, assuming a type I error of 0.05, type II error of 0.20 (power of 0.80), and 25% difference in prevalence of risk factors among anemic and nonanemic women, we needed 500 women in each group. In the first stage a complete list of clusters (cluster defined as catchment area surrounding a primary health care center (PHCC)) was formulated. Five clusters were randomly selected from each of the five administrative regions of Riyadh city, so in total there were 25 clusters. Assuming that an average Saudi family in Riyadh region will have at least one child bearing age woman in their house, 45–50 houses were randomly selected from each cluster. If more than one eligible woman present in a house, all were invited to participate and visit the PHCC. Consent was taken after explaining the objectives of the study. Inclusion criteria were; nonpregnant, age between 15 and 49 years, understanding and speaking Arabic, and resident of Riyadh city, Saudi Arabia. 1500 women were initially enrolled out of which 71 (5%) were excluded as they were either pregnant or lactating.  Figure 1 is representing the flow chart on the number of child bearing age women enrolled in the study.

### 2.3. Tool for Data Collection

Specific questionnaire was developed in Arabic to satisfy the study objectives. It inquired about sociodemographic characteristics (age, education, occupation, and type of resident); obstetric and gynecological history (menstrual history including frequency, duration and flow during menstrual cycle, contraceptive use and its duration, obstetric history on abortions, pregnancies, number of children, birth interval, and hysterectomy); family history of iron deficiency anemia and smoking history; dietary factors (inquired about regular (at least 5 days per week) intake of red meat, vegetables, eggs, tea or coffee, fresh juices, beverages, and diluted yogurt); and past medical conditions associated with anemia (peptic ulcer, hemorrhoids, cancer, bilharzias, personal and family history of iron deficiency anemia, and blood transfusion). Two female nurses were trained to conduct the interviews and take anthropometric measurements. Physician diagnosed past medical history was inquired. Responses were based on woman's verbal reply. For nutritional status assessment regular dietary frequency was requested for the last week prior to interview. Body mass index (BMI) was calculated using formula weight in kg/height in meter^2^, and the number of women belonging to following categories was calculated: underweight <18.5 kg/m^2^, normal >18.5–24.9 kg/m^2^, overweight ≥25–29.9 kg/m^2^, and obese ≥30 kg/m^2^.

### 2.4. Blood Sample for Hemoglobin Estimation

5 cc venous blood sample was taken for complete blood count including hemoglobin, red blood cells, hematocrit, and red cell indices, for example, mean cell volume, mean cell hemoglobin, mean cell hemoglobin concentration, and red blood cells distribution width. After collection blood was immediately transferred into labeled and prepared tubes containing EDTA or heparin. These were sent to the laboratory where hemoglobin estimation was performed by the laboratory technicians using UniCel Dxlt 800, Coulter cellular analysis system (Beckman Coulter Inc., USA). Light scatter method was adopted to measure hemoglobin and red cell indices under the supervision of a hematologist. Quality control procedures (Coulter 6C Cell Control) as instructed by the manufacturer were followed. Anemia was defined according to World Health Organization (WHO) hemoglobin level of <12 g/dL [3].

### 2.5. Statistical Analysis

Data were analyzed using SPSS computer statistical software package (IBM SPSS statistics version 19). The outcome variable was dichotomized as anemic and nonanemic based on WHO cutoff of <12 g/dL. Descriptive statistics with mean, standard deviation, or proportions were calculated. Age and BMI were taken as continuous, whereas number of children, birth interval, menstrual flow, dietary habits and disease status were taken as binary variables. The level of statistical significance was considered as *P* < 0.05. Univariate analysis was done to identify significant biological plausible variables. Multiple logistic regression was performed using enter method to identify important factors associated with anemia among child bearing age women. 

## 3. Results and Discussion 

The mean hemoglobin of 969 women was 12.35 (±1.80) g/dL, 95% CI 12.24–12.46 and interquartile of range 1.9.  Table 1 is presenting the mean, minimum, maximum, and interquartile range for mean corpuscular volume (MCV) [fL], mean corpuscular hemoglobin (MCH) [pg] and mean corpuscular hemoglobin concentration (MCHC), and red blood cell width (Rdw) of 969 women. Mean (±SD) value for MCH, MCV, MCHC and RDW were 79.21 (±12.17) fL, 26.37 (±6.21) pg, 32.36 (±4.91) g/dL and 14.84 (±4.65)%, respectively. Although we did not measure the gold standard serum ferritin levels, low mean levels of these indices point towards iron deficiency anemia (IDA). IDA is common in Saudi Arabia ranging from 30% to 56% [17]. Study conducted by AlQuaiz et al. found MCV, MCH, and RDW as reliable, cost-effective, and useful parameters to detect iron deficiency anemia in child bearing age women in absence of serum ferritin level [18]. 

The mean hemoglobin in anemic group and normal group was 10.81 (±1.24) and 13.38 (±1.31) g/dL, respectively (*P* < 0.01). We found 40% (390) child bearing age women suffering from anemia. These findings are consistent with other local and regional studies [1–3]. In fact the figure of 40% is higher than the previous WHO estimates and according to international definition countries with ≥40% prevalence [3] belongs to the group of countries with anemia as a severe public health problem rather than moderate or mild. Fortunately, the figures for very severe and severe anemia are much less. We found 1% (3), 2% (9), 14% (54), and 83% (322) suffering from very severe (<4 g/dL), severe (<8 g/dL), moderate (<10 g/dL), and mild (<12 g/dL) anemia, respectively. Prevention in the early mild stages can help in reducing future burden of moderate and severe anemia. Characteristics of women participating in the study are presented in Table 2.

Table 3 is representing the unadjusted odds ratio of significant variables. Among significant factors we found increasing BMI (OR 0.97, 95% CI 0.95, 0.98), absence of clots (OR 0.65, 95% CI 0.47, 0.89), absence of flooding during menstruation (OR 0.69, 95% CI 0.48, 0.96), number of children (OR 1.52, 95% CI 1.02, 2.23), birth interval (OR 2.23, 95% CI 1.20, 4.41), not eating red meat (OR 1.28, 95% CI 1.02, 1.66), not drinking diluted yogurt (OR 1.30, 95% CI 1.01, 1.69), and presence of personal (OR 2.74, 95% CI 1.79, 4.19) and family history of IDA (OR 2.55, 95% CI 1.63, 3.98) associated with anemia (Table 4). No significant association was found with sociodemographic variables, age at menarche, contraceptive use, juices, egg, vegetables, tea or coffee, peptic ulcer, hemorrhoid, and previous history of blood transfusion or hereditary blood disorders.

Multivariate logistic regression analysis revealed that infrequent (≤2 times per week) intake of meat (OR 1.59, 95% CI 1.08–2.48) was associated with anemia (Table 4). Healthy and correct diet prevents anemia, especially in child bearing age women. Dietary iron comprises of heam and nonheam iron. Heam iron is mainly available through meat intake and may be absorbed up to 50%, whereas nonheam iron mainly available through fruits, vegetables, dairy products (milk, butter, yogurt) is variable and depends upon enhancers and inhibitors for iron absorption. Absorption of nonheam iron increases in presence of meat, poultry, sea food, and juices with vitamin C, whereas tea, coffee, and egg yolk act as inhibitors towards iron absorption [WHO] [19, 20]. 

We found that those having high BMI are protective and have 5% less risk of developing anemia (OR 0.95; 95% CI 0.92–0.97) as compared to those with low BMI. Our findings support that good diet intake improves the energy supply and provides the body with nutrients making it strong and resistant against acquiring diseases [21]; however high, caloric intake does not ensure micronutrient deficiency, and people with high BMI may suffer from anemia. Strong association was found with family history of iron deficiency anemia (OR 2.64, 95% CI 1.27–5.09). If anemia is diagnosed even in a single family member, all other members should undergo screening to identify the cause and prevent future grave consequences [3]. 

We ran a second model with women (*n* = 466) having children (not shown). Women having 5 or more children were almost twice at risk of developing anemia as compared to women having <5 children (OR 1.85 (95% CI 1.03–3.54)). Number of children and birth intervals are established risk factors for anemia [22–25]. Our study found moderate but insignificant negative correlation between number of children and birth interval. During pregnancy and delivery the iron stores are depleted and women do require additional iron supplements to cover the body and fetus needs [1, 3]. It is essentially required that iron and other micronutrients are replenished accordingly. We found more the number of children, shorter the birth intervals (<1 year), hence depriving the woman's body of an appropriate interval during which it can revive its baseline energy stores level [3].

 Our study has some limitations. Around 30% of women visiting the PHCC and filling the questionnaire refused to give the blood sample for hemoglobin mainly due to fear of getting a needle prick. However, the characteristics of those women who refused to give blood sample were not different from those who gave blood sample, and we are justified in stating that it was a representative sample. Information bias may be present as past family and personal history of iron deficiency anemia was based on patient's verbal response. In addition, it is possible that residual uncontrolled confounding may have played a role in our findings.

 Current study found higher prevalence of anemia in child bearing age women than by previous studies. These findings may enhance Government efforts in preventing this disease. Mass interventions through large-scale community and primary care health programs focusing on awareness of proper and healthy diet and reproductive health will help in reducing burden of anemia. Further studies are needed to explore the specific type of anemia and interventional studies to study the effect of different dietary supplements on iron stores.

## Figures and Tables

**Table 1:** Mean blood hemoglobin and red blood cell parameters in child bearing age women in Riyadh, Saudi Arabia (*n* = 969).

Variables	Mean (SD)	Normal range	Minimum value	Maximum value	Interquartile range
Hemoglobin level (g/dL)	12.35 (±1.80)	12.0–16.0	5.2	24.4	1.9
Mean corpuscular volume (fL)	79.21 (±12.17)	80–94	18.30	110.30	10.1
Mean corpuscular hemoglobin (pg)	26.37 (±6.21)	27–32	13.10	52.30	4.3
Mean corpuscular hemoglobin concentration (g/dL)	32.36 (±4.91)	32.0–36.0	12.30	53.20	3.85
Red cell distribution width (%)	14.84 (±4.65)	11.5–14.5	3.2	45	2.2

**Table 2:** Characteristics of child bearing age women in Riyadh, Saudi Arabia.

Characteristic	Number of participants *n* (%)
Age	
Mean (SD) range: 15–49	29.05 (±10.14)
Education	
University	166 (17.1)
Secondary	212 (21.9)
Intermediate	196 (20)
Primary	177 (18.4)
Illiterate	218 (22.6)
Marital status	
Single	425 (43.9)
Married	499 (51.8)
Widow	29 (2.8)
Divorce/separated	16 (1.6)
Occupation (*n* = 804)	
Office employee	102 (14)
Student	210 (28.6)
House wife	492 (57.4)
Body mass index (*n* = 928)	
Normal (18.5–24.9 kg/m^2^)	331 (35.7)
Underweight (<18.5 kg/m^2^)	54 (5.8)
Overweight (≥25–29.9 kg/m^2^)	241 (26)
Obese (≥30 kg/m^2^)	302 (32.5)
*Menstrual history *	
Frequency of menstruation (*n* = 899)	
Once per month	865 (89.2)
Twice or more per month	34 (3.5)
Duration of menstruation (*n* = 822)	
Normal duration (5–7 days)	773 (94)
≥8 days per month	49 (6)
Passing clots during menstruation (*n* = 864)	
No	737 (76.5)
Yes	227 (23.5)
Heavy flow of menstrual blood (*n* = 864)	
No	807 (83.7)
Yes	157 (16.3)
Obstetric history	
Number of children (*n* = 466)	
Children 1–5	310 (66.5)
Children >5	156 (33.5)
Birth interval (*n* = 431)	
>1 year	364 (84.5)
≤1 year	67 (15.5)
Use of oral contraceptive pills (*n* = 624)	
No	430 (69)
Yes	194 (31)
Duration of oral contraceptive use (*n* = 194)	
<2 years	114 (58.7)
>2 to <5 years	59 (28)
>5 years	21 (10)
Use of intrauterine contraceptive device (IUCD) (*n* = 509)	
No	447 (87.8)
Yes	62 (12.2)
*Dietary factors *	
Red meat (*n* = 964)	
Yes	514 (53.3)
No	450 (46.7)
Vegetables	
Yes	626 (64.7)
No	343 (35.3)
Tea and coffee	
Yes	264 (27.4)
No	700 (72.6)
Juices	
Yes	220 (21.6)
No	748 (77.4)
Soft drinks (*n* = 964)	
Yes	363 (37.7)
No	601 (62.3)
Diluted yogurt (lasi)	
Yes	521 (54)
No	443 (46)
Eggs	
Yes	430 (44.4)
No	539 (55.6)
*Disease status *	
Peptic ulcer	
No	931 (96.6)
Yes	33 (3.4)
Hemorrhoids (*n* = 964)	
No	916 (95)
Yes	48 (5)
Cancer (*n* = 964)	
No	950 (98.5)
Yes	14 (1.5)
Bilharzias	
No	952 (98.8)
Yes	12 (1.2)
Blood transfusion	
No	950 (98.5)
Yes	14 (1.5)
Past history of hereditary blood disorder	
No	955 (99.1)
Yes	9 (0.9)
Family history of hereditary blood disorder	
No	954 (99)
Yes	10 (1.0)
Past history of iron deficiency anemia	
No	863 (89.5)
Yes	101 (10.5)
Past family history of iron deficiency anemia	
No	874 (90.7)
Yes	90 (9.3)

**Table 3:** Univariate analysis showing association of sociodemographic, gynecological/obstetrics, dietary and health status variables with anemia in child bearing age women, Riyadh, Saudi Arabia.

Variables	Anemic *N* = 390 (40%)	Normal *N* = 579 (60%)	UOR (95% CI)
BMI			
Mean (SD)	26.54 (±6.87)	28.08 (±9.37)	0.97 (0.95–0.98)
*Gynecological/obstetric factors *			
Clots during menstruation			
No	314 (81)	423 (73.4)	1.00
Yes	74 (19)	153 (26.6)	0.65 (0.47–0.89)
Flooding during menstruation			
No	336 (86.6)	471 (82)	1.00
Yes	52 (13.4)	105 (18)	0.69 (0.48–0.92)
Number of children			
<5	114 (61)	196 (70.3)	1.00
≥5	73 (39)	83 (29.7)	1.52 (1.02–2.23)
Birth interval			
>1 year	106 (79.7)	184 (89.8)	1.00
≤1 year	27 (20.3)	21 (10.2)	2.23 (1.20–4.41)
*Dietary factors *			
Red meat			
Yes	194 (49.7)	324 (56)	
No	196 (50.3)	255 (44)	1.28 (1.02–1.66)
Laban (lasi)			
Yes	225 (58)	296 (51.5)	
No	163 (42)	280 (48.6)	0.77 (0.59–0.99)
*Disease status *			
Bilharzias			
No	382 (98)	575 (99.3)	1.00
Yes	8 (2)	4 (0.7)	3.01 (0.90–10.06)
Past history of IDA*			
No	327 (83.8)	541 (93.4)	1.00
Yes	63 (16.2)	38 (6.6)	2.74 (1.79–4.19)
Family history of IDA*			
No	335 (86)	544 (94)	1.00
Yes	55 (14)	35 (6)	2.55 (1.63–3.98)

*Iron deficiency anemia.

**Table 4:** Multivariate logistic regression showing adjusted odds ratio between family history of iron deficiency anemia, infrequent intake of meat, and body mass index with anemia in child bearing age women in Riyadh, Saudi Arabia.

Variable	Adjusted odds ratio	(95% CI)	*P* value
Family history of iron deficiency anemia			
No	1.00 (reference)		
Yes	2.91	1.78–4.76	<0.01
Meat intake			
Frequent intake of meat/week	1.00 (reference)		
Infrequent intake of meat/week	1.54	1.15–2.05	<0.01
Body mass index (kg/m^2^)	0.95	0.92–0.97	<0.01
